# Figure of Image Quality and Information Capacity in Digital Mammography

**DOI:** 10.1155/2014/634856

**Published:** 2014-05-08

**Authors:** Christos M. Michail, Nektarios E. Kalyvas, Ioannis G. Valais, Ioannis P. Fudos, George P. Fountos, Nikos Dimitropoulos, Grigorios Koulouras, Dionisis Kandris, Maria Samarakou, Ioannis S. Kandarakis

**Affiliations:** ^1^Department of Biomedical Engineering, School of Technological Applications, Technological Educational Institution of Athens, Egaleo, 12210 Athens, Greece; ^2^Department of Computer Science, University of Ioannina, 45110 Ioannina, Greece; ^3^Delta Digital Imaging Centre, 6 Semitelou Street, 11528 Athens, Greece; ^4^Department of Electronic Engineering, School of Technological Applications, Technological Educational Institute (TEI) of Athens, Egaleo, 12210 Athens, Greece; ^5^Department of Energy Technology Engineering, School of Technological Applications, Technological Educational Institute (TEI) of Athens, Egaleo, 12210 Athens, Greece

## Abstract

*Objectives*. In this work, a simple technique to assess the image quality characteristics of the postprocessed image is developed and an easy to use figure of image quality (FIQ) is introduced. This FIQ characterizes images in terms of resolution and noise. In addition information capacity, defined within the context of Shannon's information theory, was used as an overall image quality index. *Materials and Methods*. A digital mammographic image was postprocessed with three digital filters. Resolution and noise were calculated via the Modulation Transfer Function (MTF), the coefficient of variation, and the figure of image quality. In addition, frequency dependent parameters such as the noise power spectrum (NPS) and noise equivalent quanta (NEQ) were estimated and used to assess information capacity. *Results*. FIQs for the “raw image” data and the image processed with the “sharpen edges” filter were found 907.3 and 1906.1, correspondingly. The information capacity values were 60.86 × 10^3^ and 78.96 × 10^3^ bits/mm^2^. *Conclusion*. It was found that, after the application of the postprocessing techniques (even commercial nondedicated software) on the raw digital mammograms, MTF, NPS, and NEQ are improved for medium to high spatial frequencies leading to resolving smaller structures in the final image.

## 1. Introduction

Full field digital mammography (FFDM) systems based on an indirect conversion detector have generally poorer response, in terms of spatial resolution, with respect to detectors based on a direct conversion process [1]. This is mainly caused by the blurring introduced by the phosphor, where X-rays are converted into light. An advantage of a digital image is the capability of software image postprocessing (application of software filters, etc.), which may help the radiologist to better visualize anatomical structures of the breast. Postprocessing techniques may be available in dedicated or general use available software packages [2–7]. General Electric has developed dedicated software named “Fine View” and “Premium View” (GE Healthcare Milwaukee, WI) to restore the loss in spatial resolution [8]. However, postprocessing techniques alter the quality characteristics of the original image in a contradicting manner [6]. That is, an enhancement in resolution or contrast may result in noise increase or add artifacts. Therefore, the radiologist should be well aware of the interpretation of post processed images. In current literature the influence of post processing dedicated software on resolution and contrast, has been investigated [6] in terms of subjective measurements via human observers [9, 10] as well as objective calculations [11, 12]. Additionally, overall image acquisition characterization has been studied through the introduction of various indexes such as the Figure of Merit (FOM) in both mammography and general radiography [11, 12] and the Global Figure of Merit (GFM) in chest radiography [13] as well as the Image Quality Indicator (IQI) in industrial radiography [14]. All these indexes characterize the raw image data in terms of noise contrast and exposure.

The aim of the present study was to demonstrate a simple method for the investigation of the influence of postprocessing techniques/algorithms on resolution and noise of digital mammography images. The method comprises spatial resolution and noise measurements in postprocessed digital images as well as the introduction of a simple figure of image quality (FIQ) combining resolution and noise in a single index. In addition, the information capacity (IC), defined within the context of Shannon's information theory, was assessed as an overall index expressing image information content. Although figure of image quality and information capacity indexes were tested on a generic software package, their utilization in characterizing image quality in dedicated medical image software will be useful.

To our knowledge, these two indexes, that is, the figure of image quality and the information capacity, providing a complete and easy way to implement assessment of the overall performance of a digital imaging system, have not been previously applied in digital mammography imaging. The application of such indexes in dedicated medical image processing and enhancement software might provide an alternative method for characterizing postprocessed medical image quality.

## 2. Materials and Methods

### 2.1. Theory

#### 2.1.1. Spatial Resolution and Signal Power Spectrum

For mammography, details of microcalcifications and fibrous spiculations radiating from lesions must be visible. It is generally believed that it is necessary to visualize structural details as small as 100 *μ*m or less [15]. It is customary to characterize spatial resolution of medical imaging systems using the Modulation Transfer Function (MTF). Use of the MTF concept requires that the imaging systems are linear stationary systems (i.e., Point Spread Function (PSF) and Line Spread Function (LSF) are independent of position in the image). MTF can be assessed by employing Coltman's formula [16, 17] which converts the square wave Contrast Transfer Function (CTF) to its equivalent sine wave MTF. CTF is given as [18]

(1) $$\begin{array}{c} \text{CTF}\left(f\right)=\frac{\left(I_{\max}\left(f\right)-I_{\min}\left(f\right)\right)}{\left(I_{\max}\left(f\right)+I_{\min}\left(f\right)\right)}, \end{array}$$

where *I*_max⁡_(*f*) is the local maxima and *I*_min⁡_(*f*) is the local contrast minima for a given frequency *f*. Given the* CTF*, the Coltman formula to determine MTF is

(2) MTF(f)=π4[C(f)+(C(3f)3)−(C(5f)5)+(C(7f)7)+⋯],

where MTF(*f*) is the sine wave MTF and *C*(*f*) is the bar target CTF. The latter gives the MTF as a function of the square wave response function (SWRF) [19]. MTF may be also used to define the detector Signal Power Spectrum (SPS). The latter is the product of the squared MTF, the squared signal, or detector response (*G*), where *G* is the gain factor (in digital units per *μGy*) [20, 21], that is, the slope of the characteristic curve, relating the mean pixel value to the incident exposure and a constant *k* which is 1.07*mGy* [22]:

(3) $$\begin{array}{c} \text{SPS}\left(f\right)=k\cdot {\left(\text{MTF}\left(f\right)\cdot G\right)}^{2}. \end{array}$$

#### 2.1.2. Noise

Noise limits small detail visualization, since it influences the high spatial frequency content of images [23]. It may be evaluated in both space and spatial frequency domain. In the space domain, noise was assessed by the measurement of a single index parameter, such as the coefficient of variation (CV), defined in terms of standard deviation (*σ*) and the mean pixel value (MPV). In the spatial frequency domain, noise was assessed via the noise power spectrum (NPS) [23–30]. *σ* and CV of image pixels were calculated in a region of interest (ROI) of 100 pixels [31] as

(4) $$\begin{array}{c} \text{CV}=\left(\frac{\sigma }{\text{MPV}}\right). \end{array}$$

Noise may be also estimated in the Fourier domain by the noise power spectrum (NPS), defined as the Fourier transform (FT) of the autocorrelation function [25]. In digital systems, the continuous function *p*(*x*) (where *p*(*x*) is the difference between the average image signal and the signal at point at *x*) of the spatial coordinates is sampled at regular intervals (Δ*x*). Thus, *p*(*x*) is evaluated at a set of discrete locations, *x* = *n*Δ*x*, *n* = 0,1, 2,…, *N*, where *X* = *N*Δ*x*. Correspondingly, functions of the spatial frequency variable *f*_*x*_ are now evaluated at discrete frequencies given by *f*_*x*_ = *k*Δ*x*, *k* = 0, ±1, ±2,…. The maximum spatial frequency sampled, the Nyquist frequency, is *f*_*x*^*N*^_ ≡ 1/(2Δ*x*). To experimentally estimate the digital noise power spectrum, the above continuous expressions must be rewritten in terms of discrete variables. With the preceding definitions, in the two-dimensional (2D) NPS defined in (5), *N* represents number of samples (*Nx* and *Ny* are the *x* and *y* dimensions of each ROI or the numbers of pixels, along the two dimensions, contained in the ROIs) and the variable *f*_*x*_ now takes on the discrete values: 0, ±1/*X*, ±2/*X*,…NPS is then found by ensemble averaging of the discrete Fourier transform of the average signal variation in the *x* and *y* directions scaled by the pixel size and the number of pixels under consideration obtained from a series of uniformly irradiated images:

(5) NPS(fx,fy) =〈|FFT  (∑nx=0Nx−1∑ny=0Ny−1p(nxΔx,nyΔy))|2〉NxNyΔxΔy,

where 〈 〉 stands for ensemble average. If the system is linear, or has been linearized (as in our case), the previous expression gives the NPS in an absolute scale (mm^2^) [24, 25, 27–30]. The normalized noise power spectrum (NNPS) is often used to compare the noise properties of different imaging systems [30]. NNPS [29] is defined as

(6) $$\begin{array}{c} \text{NNPS}\left(f\right)=\frac{\text{NPS}\left(f\right)}{{\left(G\cdot X\right)}^{2}}, \end{array}$$

where *X* is the air Kerma in *μGy*.

#### 2.1.3. Noise Equivalent Quanta (NEQ)

The combined effects of signal and noise, in terms of spatial frequency, can be expressed by the noise equivalent quanta (NEQ). The latter provides an index of the signal-to-noise ratio (SNR) associated with the diagnostic value of a medical image [29, 32, 33]. NEQ can be written as follows [32, 34–36]:

(7) $$\begin{array}{c} \text{NEQ}\left(f\right)=\frac{\text{SPS}\left(f\right)}{\text{NPS}\left(f\right)}. \end{array}$$

#### 2.1.4. Figure of Image Quality (FIQ)

**Figure 1:**
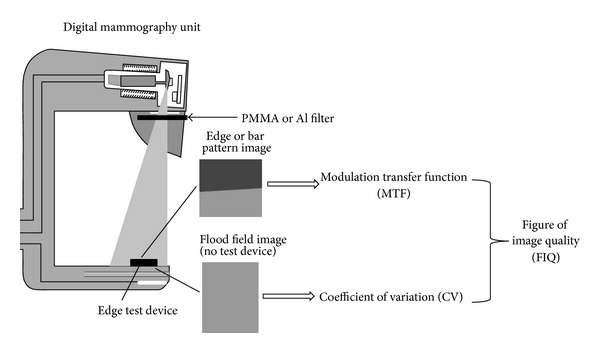
Schematic representation of the figure of image quality (FIQ) measurement.

In order to take into account the effects of postprocessing techniques in both resolution and noise, a figure of image quality (FIQ) was introduced, incorporating both factors (Figure 1). The proposed FIQ was defined as follows:

(8) $$\begin{array}{c} {\text{FIQ}}_{T}=\frac{\sum_{f=0}^{\max}\text{MTF}\left(f\right)}{\text{CV}}, \end{array}$$

where the subscript “*T*” denotes that summation is performed over the total spatial frequency range (up to the Nyquist frequency). This FIQ_*T*_ correlates the effects of all postprocessing techniques, aiming to raise detectability, suppress noise, or both [6], in a single index. High FIQ values indicate more effective postprocessing techniques in terms of resolution and noise. In addition, since postprocessing software algorithms affect differently the spatial frequency ranges (low, medium, and high), the summation over the frequencies, in (8) can be applied over a specific frequency region. In this manner, a FIQ_*R*_ equals

(9) $$\begin{array}{c} {\text{FIQ}}_{R}=\frac{\sum_{f=f1}^{f2}\text{MTF}\left(f\right)}{\text{CV}} \end{array}$$

and can be used to characterize the postprocessing algorithm over a specific frequency region. The subscript “*R*” in (9) stands for spatial frequency range.

#### 2.1.5. Information Capacity (IC)

**Figure 2:**
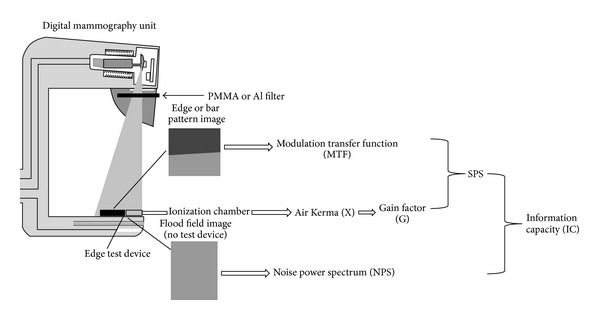
Schematic representation of the information capacity (IC) measurement.

The concept of image information capacity (IC) was been introduced within the context of Shannon's information theory, in order to assess image information content [20, 24, 37–45] (see the Appendix and Figure 2). However, little work relevant to medical imaging has been published up till now [20, 40–46]. According to Shannon's theory, the image information capacity, per unit of image area, is given by ((A.1)) (in the Appendix). For the detectors used in both conventional and digital X-ray imaging, power spectra can be represented as one-dimensional functions of spatial frequency, SPS, and NPS due to rotational symmetry [24]. The information capacity is then given by [40, 42–45, 47–49]

(10) $$\begin{array}{c} \text{IC}=\pi \overset{\infty }{\underset{0}{\int }}\text{lo}{\text{g}}_{2}\left[1+\left(\frac{\text{SPS}\left(f\right)}{\text{NPS}\left(f\right)}\right)\right]f\;df. \end{array}$$

### 2.2. Experiments and Calculations

The relationship between the mean pixel value and the air Kerma incident on the detector can be plotted by measuring the detector response as follows. A phantom of Polymethyl Methacrylate (PMMA) with total thickness of 25 mm, to simulate spectrum alteration by a small breast, was positioned at the tube exit port of a GE Essential digital mammographic unit (incorporating a Cesium Iodide doped with Thallium (CsI : Tl) scintillator coupled to a Thin Film Transistor (TFT) array of 100 *μ*m pixel size and 19 × 23 cm field of view) and exposed using a tube voltage of 28 kV [49]. A mammographic ion chamber (Victoreen Model 6000-529, chamber volume of 3.3 cm^3^) combined with a Victoreen Model 4000M+ dosimeter was positioned at the surface of the breast support table with the grid removed and the entrance surface air Kerma measured for a range of tube current-time products. The readings were corrected to the surface of the imaging detector using the inverse square law. It was determined that the imaging detector has a distance of 66 cm from the tube focus, using data provided by the manufacturer. The images were saved as both raw data and processed with “fine view.” Afterwards, files were transferred to the workstation for analysis. 100 × 100-pixel square ROIs were measured from each image. The mean pixel value and the standard deviation within that region were measured. The system's response curve was fitted using a linear equation of the form MPV = *a* + *b* · *X*, where *a* and *b* are adjustable coefficients and *X* represents the entrance air Kerma at the detector surface plane. In this manner, the relationship between MPV and the detector entrance surface air Kerma was determined. Then from the slope of this curve, the value of the gain factor (*G*) was found. The magnitude of the pixel offset at zero air Kerma was estimated [50]. MTF was experimentally determined by the SWRF method. A Nuclear Associates resolution test pattern (typ-53, Nuclear Associates), containing lead (Pb) lines of various widths corresponding to various spatial frequencies (from 0.25 mm^−1^ to 10 mm^−1^), was used to obtain pattern images [51]. The MTF test pattern was irradiated by X-rays using a molybdenum (Mo/Mo) target/filter combination at 28 kVp and 10 mAs. The test pattern images were obtained in DICOM format. MTF curves were calculated from the density variations (digital SWRF) in digital images (both raw and filtered). The latter were obtained across vertical directions with respect to the test pattern lines, employing Coltman's formula in (2) [52, 53]. SPS was calculated from (3) using MTF data and the gain factor determined from the detector response curve. Pattern images were also analyzed using Adobe Photoshop professional Version 8 image analysis software.

The method was applied in a digital mammogram, which was postprocessed by both “fine view” and three sharpening filters, incorporated in a commercial image processing tool (Photoshop Version 8, Adobe Systems Incorporated, 345 Park Avenue, San Jose, California, USA). This version of Photoshop incorporates three basic sharpening filters hereafter referred to as: (a) “sharpen,” (b) “sharpen more,” and (c) “sharpen edges.” The three sharpening filters were then applied on the raw image data and three new images of the MTF test pattern were obtained. NPS was calculated from uniformly exposed images. The available homogeneously exposed regions were covered with overlapping square ROIs of 128 × 128 pixel. For each of these squares, the 2D NPS was calculated according to (5). The data from the 2D NPS were then rearranged in a single one-dimensional (1D) vector as functions of their spatial frequency. The 1D NPS curves were then interpolated linearly to obtain a constant interval sampling. Normalized NPS was calculated according to (6). Finally, noise equivalent quanta and information capacity were calculated according to (7) and (10) by using MTF, NPS, and system gain data. The use of these two software packages was chosen, in order to validate the possible generic use of the introduced FIQ [6, 10, 19, 54, 55].

## 3. Results

**Figure 3:**
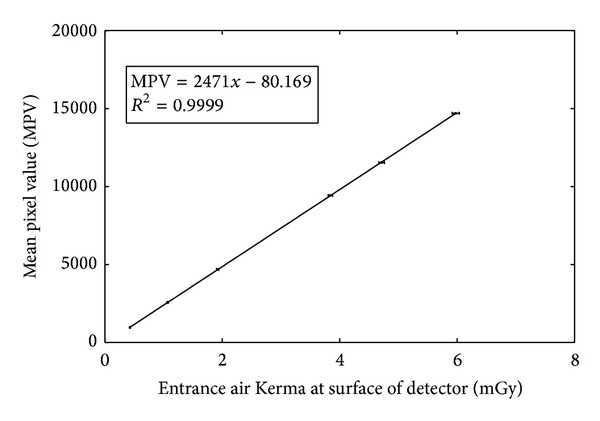
Detector response (pixel value versus detector entrance air Kerma).

Figure 3 shows the detector response curve of the digital mammographic unit at 28 kV. The detector was found to have a linear response covering the whole exposure range, with a pixel value offset of 32. The linear no-threshold fit gave a correlation coefficient (*R* ^2^) greater than 0.9999. There was no relative deviation between any of the experimental points, used to generate the fitted curves, and the corresponding fitted curve, over the whole exposure range. The gain factor *G* was determined as the slope of the characteristic curve, relating the mean pixel value to the incident exposure. Using flat-field images, the gain factor was determined by linear regression to be *G* = 2471 ± 4.287 digital units per *μGy*.

**Figure 4:**
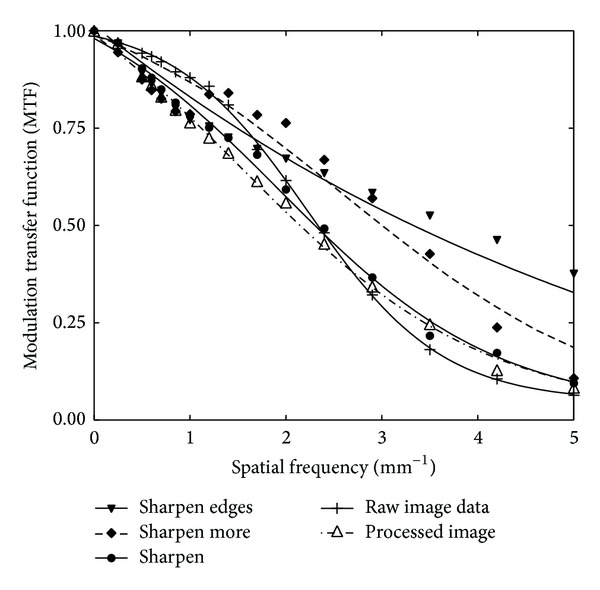
Comparison of the MTFs of the raw, “fine view” processed, and “filtered” image data (data obtained from [7]).

Figure 4 shows MTFs of the raw, “fine view” processed, and “filtered” image data. “Fine view” processed image data refer to the raw data subject to filtration by the mammographic unit workstation. The relatively lower MTF values corresponding to the raw image data could be attributed to the routine clinical practice conditions followed for this study, for example, grid, bucky, compression plate, and PMMA slabs in place. MTF values of the “fine view” processed image show a tendency to increase at spatial frequencies above 2.7 mm^−1^. On the other hand, in the low frequency range, MTF appears decreased. The overall MTF curve of the “fine view” processed image data has lower slope than the raw data. This provides a small boost to the higher frequencies, giving the opportunity to increase the visibility of small objects in the final image. The “sharpen” filter shows reduced MTF values with respect to the aforementioned filter for spatial frequencies up to 2.8 mm^−1^. Beyond this point, the “sharpen” filter results in additional increase of the MTF of the raw data, giving better resolution in this frequency range. An increase in the MTF was provided by the other two filters. The “sharpen more” filter raises MTF values compared to those of the raw data, at frequencies above 1.4 mm^−1^. Similarly, the “sharpen edges” filter was found to raise MTF at frequencies above 1.7 mm^−1^. The “sharpen more” filter follows a sigmoidal MTF pattern similar to that of the raw image data. It gives a MTF increase in the medium to high spatial frequency region (*f* = 1.2 to *f* = 5 mm^−1^). The “sharpen edges” filter shows a tendency to “linearise” the “sigmoidal” shape of the raw MTF data providing an increase, beyond the spatial frequency of 2.9 mm^−1^, as compared to all the aforementioned filters and the raw image data.

To characterize the filters employed, we have used a reverse engineering methodology that uses image difference metrics and histogram normalization [56]. It was applied to the four sharpening tools involved in the specific experimental set-up and we have determined that, in this domain, “fine view” behaves as an adaptive contrast enhancement filter (see [57]). The “sharpen” filter behaves as a simple local sharpening filter with kernel [0 −1 0, −1 1+4*α* −1, 0 −1 0], where *α* ranges within [0.7, 1]. This is a sharpening filter that is derived by adding to the original image the result of an unscaled square 3 × 3 difference operator (high pass filter). The “sharpen more” filter shows similar behavior as the “sharpen” one, but it increases contrast by 50% in average (and thus noise) than the simple “sharpen” filter: [0 −1 0, −1 1+4*α*  −1, 0 −1 0], where *α* ranges within [1.0, 1.3]. The “sharpen edges” employs a simple Laplacian edge detector [−1−1−1, −18−1, −1−1−1] (3 × 3 square) that is then rescaled so that intensity values fall within the same range as the original image. It is then thresholded and added to the original image value weighted by a factor* k* that we determined to be in the range [1.1, 1.3]. The threshold for edge sharpening is determined based on the histogram of the edges (only edges of a certain intensity are considered for enhancement).

**Figure 5:**
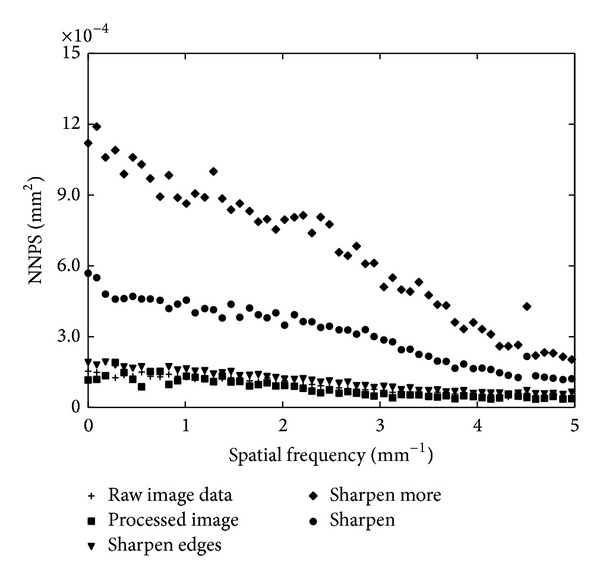
NNPS of the raw, “fine view” processed, and “filtered” image data from an exposure of 25 mm PMMA at 28 kV, Mo/Mo, 10 mAs.

Figure 5 shows the 1D NNPS for the raw, “fine view” processed and filtered images. The image filtered by the “sharpen more” filter showed higher levels of noise. Lower NNPS was obtained for the “fine view” processed image which however is very close to both the raw image and the one filtered by the “sharpen edges” filter. Raw image shows almost a constant (white) noise power spectrum showing a very small decrease with spatial frequency. This is attributed to the fact that, in the flat panel system incorporated in the GE Essential digital mammographic unit, the detector elements are fixed and a 2D correction, which reduces the structured noise, is applied for each image. In the middle to high frequency range, the noise level of the image is related to the MTF of the system. Since the MTF of the flat panel system remains high up to the Nyquist frequency, the NNPS remains quite constant over a large spatial frequency band.

The noise was also estimated by means of the coefficient of variation in an area of 100 pixels as shown in Table 1. The “sharpen edges” filter exhibits a CV of 0.630%. The image filtered by the “sharpen more” filter appeared to be noisier corresponding to a CV of 1.796%. The image filtered by the “sharpen” filter was poorer in resolution and moderate in noise with a CV of 1.186%.

**Figure 6:**
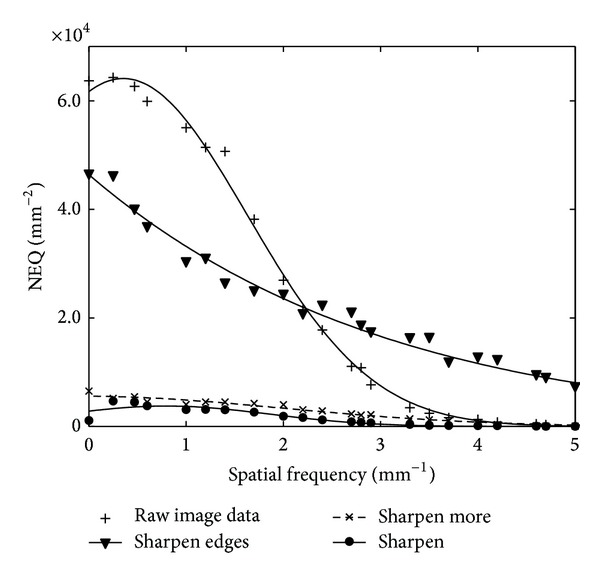
NEQ of the raw, “fine view” processed, and “filtered” image data calculated from the MTF and NPS.

Figure 6 shows noise equivalent quanta of the raw, “fine view” processed, and “filtered” image data calculated from the MTF, NPS, and the incident X-ray exposure data. The shapes of the NEQ curves are affected by both NPS and MTF. As shown in Figure 6, in the low frequency range (0–2 mm^−1^), raw image data has higher NEQ values followed by the NEQ corresponding to the “sharpen edges” filtered image. This is attributed to the low NPS and high MTF values of the raw image data in this spatial frequency range. Accordingly, low contrast objects of large dimensions may be better visualized at the unprocessed image. In the medium to high spatial frequency range (2–5 mm^−1^), NEQ of the “sharpen edges” filtered image is clearly higher than the corresponding values of the raw image data. This may be explained by the higher MTF values in this spatial frequency range. The “sharpen” and “sharpen more” filtered images have reduced NEQ values due to their high NPS caused by the noise that the filtering process introduces to the images.

In Table 2, the FIQ_*T*_ values of the three postprocessed images are tabulated. According to the FIQ_*T*_ value, the best postprocessed image, in terms of resolution and noise, is the “sharpen edges” filtered image. On the other hand, the lowest FIQ_*T*_ values were obtained for the “sharpen more” filtered image.

In Table 3, the FIQ_*R*_ for three spatial frequency regions 0–2.0 mm^−1^ (low), 2.0–5.0 mm^−1^ (medium), and 5.0–8.0 mm^−1^ (high) are demonstrated. From Table 3, the best filtering method, regarding resolution and noise, for all the spatial frequency ranges is still the “sharpen edges” filter. FIQ_*R*_ for the “sharpen more” filtered image, however, changes from one region to the other. That is, for the low and medium frequency region, the “sharpen more” software filter shows the lowest values, while, in the high frequency region, the lowest values were obtained for the “sharpen” filter. It is of importance to notice that, in some cases, the raw data showed better image quality characteristics than the postprocessed data. This is the case of the low and high frequency region where the FIQ_*R*_ corresponding to the raw data exhibits higher values than the FIQ_*R*_ corresponding to the image filtered by the “sharpen” filter. These values, as well as the data demonstrated in Figure 4 and Tables 1 and 2, are indicative of the effect that a postprocess algorithm may have on a digital image. According to these findings, the postprocessed image can be totally or partially better in certain spatial frequency regions and to show loss of information in some other frequency regions.

In Table 4, the information capacity (bits/mm^2^) values of the raw and the postprocessed data are demonstrated. The values are mainly affected by the noise equivalent quanta. The “sharpen edges” filtered image gave an IC of 78.96 × 10^3^ bits/mm^2^, which is higher than the value corresponding to the raw image data (60.86 × 10^3^ bits/mm^2^). This is due to the higher overall NEQ values of the “sharpen edges” filtered image calculated from the integral in (10). In a similar way, the information capacity values of the “sharpen” and “sharpen more” filtered images are affected by the corresponding relatively low NEQ values.

## 4. Discussion

**Figure 7:**
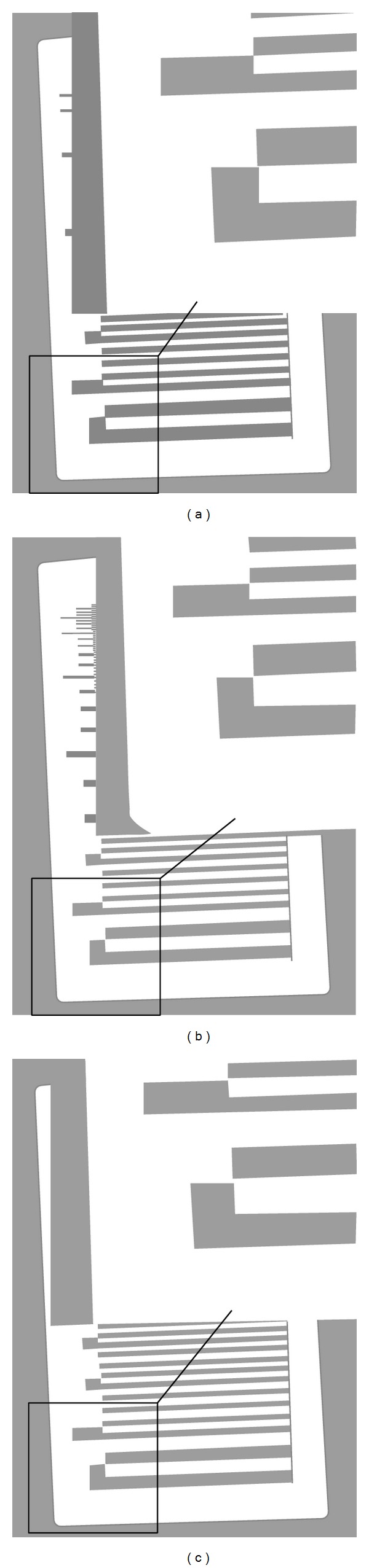
Images of the bar pattern: raw (a), sharpen edges filtered image (b), and sharpen more filtered image (c).

**Figure 8:**
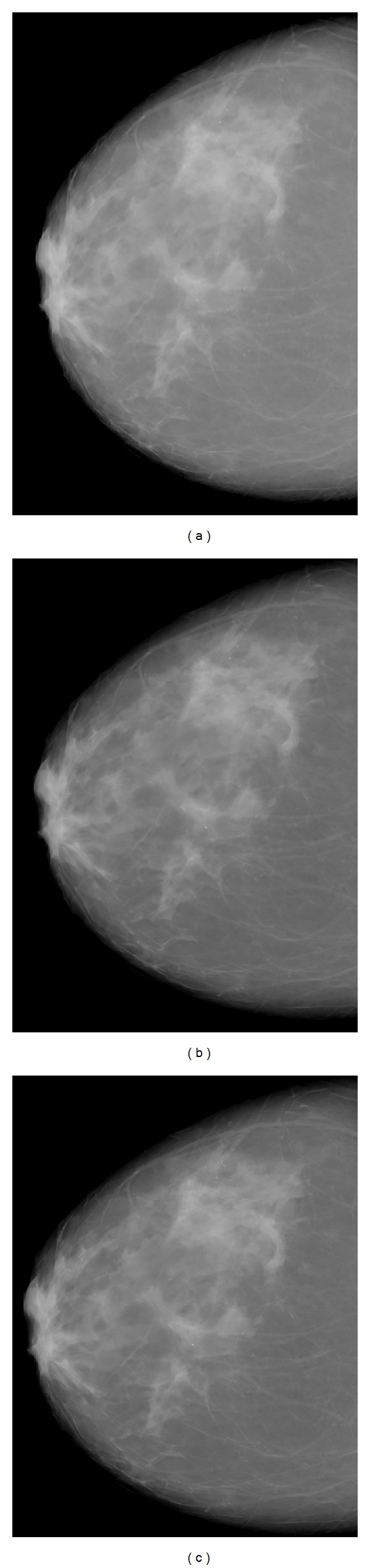
Images of a breast: raw (a), sharpen edges (b), and sharpen more (c) filtered images.

The practical value of FIQ and IC from the application point of view and clinical point of view can be depicted from Figures 7 and 8. The image shown on the left of Figure 7 is the raw image data with a corresponding cropped region of the MTF test pattern image. The image in the center (middle) has been filtered by the “sharpen edges” filter, while the image on the right has been filtered by the “sharpen more” filter. The “sharpen edges” image (FIQ_*T*_: 1906.1) appears sharper than the raw one, while no added noise is present. This is compatible with the high FIQ_*T*_ value of this image. On the other hand, the “sharpen more” filtered image (FIQ_*T*_: 643.4) appears sharper than the raw image data, however, showing a “grainy texture” due to filter's added noise, which reduces FIQ_*T*_.

Similarly to Figure 7, in Figure 8, the raw image of a breast at 29 kVp and the corresponding filtered images are shown. The filtered images were preliminary evaluated by a high experienced radiologist (N.D.) who postulated that the microcalcifications were clearly more visible in the “sharpen edges” filtered image. In particular, the “sharpen edges” filtered image shows improved overall sharpness, providing more distinctive borders in the breast structures. The above findings can demonstrate that FIQ_*T*_ can be used as an image quality index.

FIQ_*T*_ and IC do not follow the same tendency regarding the applied filter (Tables 2 and 4). According to FIQ_*T*_,the “sharpen” filtered image is better than the “sharpen more” filtered image, while, according to IC, the opposite occurs. This may be explained by the fact that IC, in addition to the MTF, is affected by the shape of the NPS curve in spatial frequency. The comparison between the FIQ_*T*_, IC, and an observer performance evaluation method (i.e., receiver operating characteristic (ROC) analysis) [58] with the use of real mammographic images or contrast detail dedicated breast phantoms is beyond the scope of this paper. Such studies, however, may be useful in translating the numerical values of FIQ_*T*_ and IC in image features detectability. The present method utilizes a bar pattern incorporating interbar distances between 100 *μ*m (10 mm^−1^) and 4 mm (0.25 mm^−1^). This method can be further utilized to other imaging modalities such as nondestructing testing techniques, as long as the above dimensions are of interest. Although figure of image quality and information capacity indexes were tested on a generic software package, their utilization for image quality characterization, in dedicated medical image software, will be useful provided there is image processing capability [59–63]. This method can be further utilized to other imaging modalities and 3D imaging modalities [64–66] considering that the images are obtained by specialized MTF phantoms, respectively [67, 68].

A limitation of this study could be that the information capacity is not expressed for specific frequency values, since it is the outcome of integrating over the spatial frequency bandwidth [51]. Thus, IC is significantly affected by the maximum frequency contained in the signal, that is, the frequency bandwidth over which integration is performed. On the contrary, FIQ_*R*_ and FIQ_*T*_ are dependent upon frequency range and the CV, making a straightforward mathematical relationship among these figures of merit difficult. Detectors with better spatial resolution properties (incorporating thinner scintillating screens or direct detectors such as amorphous selenium a-Se) exhibit a larger bandwidth, which can cause an increase in IC values. In addition to this, information capacity is calculated using spatial frequency as a factor, which multiplies the logarithmic term containing SNR. As the allowed spatial frequency region is enlarged to include higher and higher spatial frequencies, the information capacity slightly increases at fixed SNR. This affects IC in a sense that it may be preferably emphasized by high frequency information content, that is, the kind of information that is expressed by spatial resolution and sharpness, which is better displayed by thin screens.

## 5. Conclusions

In the present study, the influence of postprocessing techniques on resolution and noise in digital mammography was investigated via the introduction of a figure of image quality. In addition, image quality was assessed through the application of information capacity. As a test case, the digital image of a bar pattern obtained from a digital mammography unit was postprocessed with three sharpening filters, incorporated in a widely available image processing tool. Although the implementation of the figure of image quality and information capacity concepts was done with a commercial generic processing tool, their utilization in characterizing image quality in dedicated medical image software may be of importance. The findings of this study are indicative of the effect that a postprocess algorithm may have on a digital image. This effect can be estimated through the determination of single index parameters such as the figure of image quality and the information capacity introduced in this work, as well as by more conventional frequency dependent parameters such as MTF, NPS, and NEQ.

## Figures and Tables

**Table 1:** Noise measurement of the raw and the postprocessed data.

	Raw image	“Sharpen” filtered image	“Sharpen more” filtered image	“Sharpen edges” filtered image
*σ*	0.913	1.909	2.887	1.012
$\overset{-}{\mu }$	76.53	160.91	160.77	160.58
CV (%)	1.193	1.186	1.796	0.630

**Table 2:** FIQ_*T*_ values of the raw and the postprocessed data.

	Raw image	“Sharpen” filtered image	“Sharpen more” filtered image	“Sharpen edges” filtered image
Sum of MTF	10.82	10.41	11.55	12.01
FIQ_*T*_	907.3	877.9	643.4	1906.1

**Table 3:** FIQ_*R*_ values of the raw and the postprocessed data for low, medium, and high frequency region.

FIQ_*R*_ (mm^−1^)	Raw image data	“Sharpen” filtered image	“Sharpen more” filtered image	“Sharpen edges” filtered image
0–2.0	797.8	754.6	517.8	1425.4
2.0–5.0	148.3	163.1	154.5	516.3
5.0–8.5	18.1	18.0	19.6	130.6

**Table 4:** Information capacity values of the raw and the postprocessed data.

	Raw image data	“Sharpen” filtered image	“Sharpen more” filtered image	“Sharpen edges” filtered image
IC × 10^3^ (bits/mm^2^)	60.86	42.09	50.37	78.96

## Appendix

### Information Capacity (IC)

According to Shannon's information theory, if it is possible to distinguish *N*_*S*_ different signal intensity levels of duration *T* on a channel, we can say that the channel can transmit log_2_ *N*_*S*_ bits in time *T*. The rateof transmission is then log_2_ *N*_*S*_/*T*. More precisely, the image information capacity, per unit of image area, may be defined by (10) as follows [47]:

(A.1) $$\begin{array}{c} \text{IC}=\frac{\lim_{T\to \infty }\text{lo}{\text{g}}_{2}N_{S}}{T}=n_{p}\text{lo}{\text{g}}_{2}N_{S}, \end{array}$$

where *n*_*p*_ is the number of image elements (pixels) per unit of area that can be registered in an image element. If the transmitter has a dynamic range (*D*) and the noise in the transmission channel is assumed to be Gaussian (normal (0, *σ* ^2^)) and independent of the signal amplitude, then an expression may be derived for *N*_*s*_ in terms of *D* and *σ*. For this case, the levels are equally spaced between zero and *D* with a spacing determined by the acceptable error rate for signal transmission. If each level has an interval of =±*kσ*, according to Wagner et al. [20], then the spacing becomes 2*kσ*, and one obtains *N*_*S*_ − 1 = *D*/2*kσ*. Equation (A.1) may now be written as follows: *IC* = *n*log⁡_2_(1 + *D*/2*kσ*). Shannon showed that, in the frequency domain, there is a definition of information capacity that is consistent with the equations described above and which serves as a unique upper limit for the rate of information transmission [46]. This upper limit for the number of distinguishable signals in communication channel of bandwidth Δ*f* is defined as $N_{S}\leq {\left(\sqrt{(\text{SPS}+\text{NPS})/\text{NPS}}\right)}^{2T\Delta f}$. Consequently, the channel capacity is limited by [47]

(A.2) $$\begin{array}{c} \text{IC}=\frac{\text{lo}{\text{g}}_{2}N_{S}}{T}\leq \Delta f\text{lo}{\text{g}}_{2}\left(\frac{\left(\text{SPS}+\text{NPS}\right)}{\text{NPS}}\right), \end{array}$$

where SPS and NPS are assumed to be constant over the frequency interval Δ*f*. Wagner et al. have expressed this equation [20] under the following form:

(A.3) $$\begin{array}{c} \text{IC}\left(\Delta f\right)=2\Delta f\text{lo}{\text{g}}_{2}{\left(\frac{\left(\text{SPS}+\text{NPS}\right)}{\text{NPS}}\right)}^{1/2}, \end{array}$$

where the exponent of 1/2 is explained since the ratio of amplitudes is replaced by a ratio of powers and the factor 2Δ*f* results directly from the sampling theorem. For any function having a bandwidth of Δ*f*, sampling at intervals 1/2Δ*f* completely specifies the function. If the function is assumed to be limited to a time interval *T*, then, for a real function, there are just 2Δ*fT* independent values of 2Δ*f* values per unit time. The dimensions of IC are bits per unit time [20]. If (A.2) is integrated over the whole frequency range, then information capacity is given by

(A.4) $$\begin{array}{c} ΙC=\overset{f}{\underset{0}{\int }}\text{lo}{\text{g}}_{2}\left[1+\frac{\text{SPS}\left(f\right)}{\text{NPS}\left(f\right)}\right]df, \end{array}$$

where *f* is greater than or equal to the band limit of the signal power. The 2D form of (A.3) is given by IC = (2Δ*f*_*x*_)(2Δ*f*_*y*_)log_2_((SPS+NPS)/NPS)^1/2^ and written in terms of spatial frequency, leading similarly to an integral over the upper right-hand quadrant in *f*_*x*_, *f*_*y*_(*f* = (*f*_*x*_^2^ + *f*_*y*_^2^)^1/2^) space or using polar frequency coordinates and assuming cylindrical symmetry of SPS and NPS, with

(A.5) $$\begin{array}{c} ΙC=\frac{1}{2}\overset{f}{\underset{0}{\int }}\text{lo}{\text{g}}_{2}\left[1+\frac{\text{SPS}\left(f\right)}{\text{NPS}\left(f\right)}\right]2\pi f\;df \end{array}$$

expressed in bits per unit area [20]. For the detectors used in both conventional and digital X-ray imaging, power spectra can be represented as one-dimensional functions of spatial frequency *f*, SPS(*f*) and NPS(*f*) due to rotational symmetry [20]. Equation (A.5) now becomes (10) [40, 42–45, 47–49]. This expression for information capacity is dependent upon the dynamic range or more properly the total power available to the transmitter.
